# Case Report: Prenatal neurological injury in a neonate with pyruvate carboxylase deficiency type B

**DOI:** 10.3389/fendo.2023.1199590

**Published:** 2023-07-07

**Authors:** Mei Xue

**Affiliations:** Department of Endocrinology, Zhongnan Hospital of Wuhan University, Wuhan, China

**Keywords:** case report, pyruvate carboxylase deficiency, neurological injury, genetic analysis, prenatal diagnosis

## Abstract

**Background:**

Pyruvate carboxylase (PC) is a key enzyme for gluconeogenesis. PC deficiency (PCD) is an extremely rare autosomal recessive metabolic disease and is divided into three types. Type B PCD is clinically featured by lactic acidosis, hyperammonemia, hypercitrullinemia, hypotonia, abnormal movement, and seizures.

**Case presentation:**

Here, we report the first case of type B PCD in China, presenting with intractable lactic acidosis shortly after birth. A compound heterozygous mutation in the PC gene was identified by whole-exome sequencing, NM_001040716.2: c.1154_1155del and c.152G>A, which were inherited from her asymptomatic parents, respectively. Furthermore, prenatal neuroradiological presentations including widened posterior horns of lateral ventricles, huge subependymal cysts, and increased biparietal diameter and head circumference were concerned. Symptomatic treatment was taken and the infant died at 26 days.

**Conclusion:**

To our knowledge, this is the minimum gestational age (22w5d) that’s when the prenatal onset of the neuroradiologic phenotype of PCD was observed. PCD has a poor prognosis and lacks an effective treatment, so this paper is shared to highlight the importance of PCD prenatal diagnosis in the absence of family history.

## 1 Introduction

Pyruvate carboxylase (PC) catalyzes the conversion of pyruvate to oxaloacetate, which is a committed step of gluconeogenesis. PC deficiency (PCD) (OMIM 266150) is a rare autosomal recessive disease characterized by hyperlactatemia and acidosis. PCD has mainly been reported in European and American countries, and the estimated prevalence is 1 in 250,000 (1). However, only three cases involving two families have been described in the Chinese population (2–4).

It is generally accepted that there are three clinical types of PCD. Type A (infantile type) was found mainly in North American populations with severe symptoms appearing in infancy. Type A is featured by developmental delays, hyperlactatemia, and increased acidity in the blood which can lead to vomiting, abdominal pain, extreme fatigue, muscle weakness, and difficulty breathing. In severe cases, death occurs in infancy or early childhood (5, 6). Type B (severe neonatal type) was reported mainly in Europe. Affected infants have severe lactic acidosis, hyperammonemia, hypercitrullinemia, and liver failure. There may also be neurological disorders, including hypotonia, abnormal movement, and seizures. Patients with Type B usually survive less than 3 months after birth (7–9). Type C (intermittent/benign type) patients have normal or mild neurodevelopmental delays and intermittent metabolic acidosis (10, 11).

Here, we report a female newborn presenting with intractable lactic acidosis. She was diagnosed genetically and biochemically with type B PCD. Her mother’s obstetrical examination, including ultrasound, fetal brain magnetic resonance imaging (MRI), and amniotic fluid analysis were reviewed. Her neurological imaging abnormalities were detected as early as 22 weeks of gestation which is the smallest gestational age reported in the literature.

## 2 Case presentation

This female neonate, the first child of a non-consanguineous couple, was born at 38w6d gestational age *via* cesarean section. Her birth weight was 2.79kg, and her body length and head circumference were 47cm and 35.5cm, respectively. Apgar scores of one and five minutes were rated as 9 and 10, respectively. Five hours after birth, she was admitted to the pediatric intensive care unit due to tachypnea and dysphoria. After admission, the patient presented with convulsions and irritability.

Laboratory results (**Table 1**) revealed that the blood lactic acid was 24.8mM and ammonia was 294.4mM. Blood bilirubin increased progressively with a peak of 269.8μM. Her blood glucose was normal. Blood myocardial enzyme levels were extremely elevated, including creatine kinase at 973U/L, creatine kinase-MB at 145U/L, lactate dehydrogenase at 769U/L, α-hydroxybutyrate dehydrogenase at 581U/L. The 3-beta-hydroxybutyrate was 0.37mM and the urine ketone was 2+. Arterial blood gas analysis (**Table 2**) disclosed metabolic acidosis with pH of 7.24 and HCO_3_^−^ of 5.2mM. Inherited metabolic disease determination (**Table 1**) reported a significant increase in blood citrulline (109.4mM) and mild elevations of tyrosine (223.4mM) and alanine (408.5mM). Urine organic acids determination (**Table 1**) showed that urinary lactic acid (7262nmol/mg creatinine), 2-hydroxybutyric acid (310.9nmol/mg creatinine), pyruvate (1416.8nmol/mg creatinine), 3-hydroxybutyric acid (1725.3nmol/mg creatinine), 4-hydroxyphenyllactic acid (979.6nmol/mg creatinine) and other organic acids increased severely, which suspected the possibility of genetic metabolic disease. For this reason, Trio-whole exome sequencing (WES) was conducted (**Supplementary materials and methods**), but it took 2-3 weeks. Her chest computed tomography showed no abnormalities, but an echocardiogram revealed mild patent ductus arteriosus, patent foramen ovale, and mitral and tricuspid regurgitation.

**Table 1:** Laboratory results.

Variable	Patient’s value	Reference value
Blood routine		
White blood cells (×10^9^/L)	19.88	5-30
Red blood cells (×10^12^/L)	4.89	3.5-6.6
Hemoglobin (g/L)	173	140-200
Platelets (×10^9^/L)	190	242-378
Neutrophils (%)	66.3	31-52
Lymphocytes (%)	20.6	31-48
Biochemical test		
Glucose (mmol/L)	4.2	3.9-6.1
Lactate (mmol/L)	24.8	0.5-2.5
3-β-hydroxybutyrate(mmol/L)	0.37	0-0.28
Blood ammonia (μmol/L)	294.43	18-72
Albumin (g/L)	31.7	40-55
Alanine transaminase (U/L)	12	7-45
Aspartate transaminase (U/L)	74	13-35
Alkaline phosphatase (U/L)	187	8-57
γ-glutamyl transpeptidase (U/L)	109	30-120
Total bilirubin (μmol/L)	54.6	5-21
Direct total bilirubin (μmol/L)	5.4	0-7
Indirect total bilirubin (μmol/L)	49.2	1.5-18
Blood urea nitrogen (mmol/L)	3	2.8-7.6
Creatinine (μmol/L)	48.5	49-90
Uric acid (μmol/L)	356.8	155-357
Creatine kinase (U/L)	973	<145
Creatine kinase-MB (U/L)	149	0-25
Lactate dehydrogenase (U/L)	769	110-245
Lactate dehydrogenase-MB (U/L)	151	15-65
α-hydroxybutyrate dehydrogenase (U/L)	581	72-158
Inherited metabolic diseases determination		
Alanine (μmol/L)	408.462	70-400
Tyrosine (μmol/L)	223.422	25-200
Citrulline (μmol/L)	109.364	4-30
Urine organic acids determination		
Lactic acid (nmol/mg creatinine)	7262.01	0-4.7
Pyruvate (nmol/mg creatinine)	1416.75	0-24.1
2-hydroxybutyric acid (nmol/mg creatinine)	310.9	0
3-hydroxybutyric acid (nmol/mg creatinine)	1725.34	0-3.7
4-hydroxyphenyllactic acid (nmol/mg creatinine)	979.62	0-7
4-hydroxyphenylpyruvic acid (nmol/mg creatinine)	30.04	0-0.9
3-hydroxyglutaric acid (nmol/mg creatinine)	11.24	0
Acetylglycine (nmol/mg creatinine)	12.25	0-0.1
2-keto-isovaleric acid (nmol/mg creatinine)	10.48	0-0.1

She was supported by low-flow oxygen and some symptomatic and empirical treatments were taken. Sodium bicarbonate was used to correct metabolic acidosis, arginine to reduce blood ammonia, vitamin C and coenzyme Q10 to nourish myocardium, L-carnitine and vitamin B1 to regulate metabolic balance, blue light to treat jaundice, antibiotics to fight infection. While metabolic acidosis was gradually corrected by sodium bicarbonate at the beginning, the acidosis reappeared once no alkaline liquid was added (**Table 2**). During the 13-day hospital stay, the blood lactate levels were several times higher than normal all the time (**Table 2**). The guardian was informed of the condition and they chose palliative care. The girl was discharged with oral administration of L-carnitine, coenzyme Q10, and vitamin B1. Through the post-discharge follow-up, the mother reported dysilthia, dyskoimesis, dysphoria, and occasional convulsion. 9 days after discharge, her lactic acid was 16mM, ammonia was 87.46μM, arterial pH was 7.2 and HCO_3_^−^ was 3.7mM. On the 26th day of her life, the girl passed away at home.

**Table 2:** Blood gas results.

Age	1 day	2 days	3 days	4 days	5 days	7 days	8 days	9 days	11 days	12 days	13 days	22 days
Blood source	Arterial	Venous	Venous	Arterial	Arterial	Arterial	Arterial	Arterial	Venous	Arterial	Arterial	Arterial
Lactic acid (mmol/L)	24.8 (Venous)	21.8	6.8	8.1	–	8.5	8.3	10.2	9.2	9.9	14.7	16
pH	7.24	7.37	7.39	7.39	7.68	7.49	7.49	7.49	7.48	7.48	7.38	7.2
PO_2_ (mmHg)	143	53	48	221	218	212	209	126	48	187	115	138
PCO_2_ (mmHg)	12.4	14.1	24.9	22.6	10.3	25.3	26.5	18.9	23.3	14.3	15	9.7
HCO_3_^-^ (mmol/L)	5.2	7.9	15.0	13.5	19.7	19.5	20.2	14.3	17.3	16	8.9	3.7
Base excess (mmol/L)	-21.2	-15.2	-8.6	-10	-8.1	-3.1	-2.4	-7.9	-5.0	-12.7	-14.4	-23.7
SO_2_	98.9%	87.8%	83.9%	99.6%	99.7%	99.6%	99.6%	98.9%	87.1%	99.6%	98.4%	98.6%
Application of sodium bicarbonate	Yes	Yes	Yes	Yes	No	No	No	Yes	No	No	No	No

PO_2_, partial pressure of oxygen; PCO_2_, partial pressure of carbon dioxide; HCO_3_^-^, bicarbonate; SO_2_, oxygen saturation.

**Figure 1:**
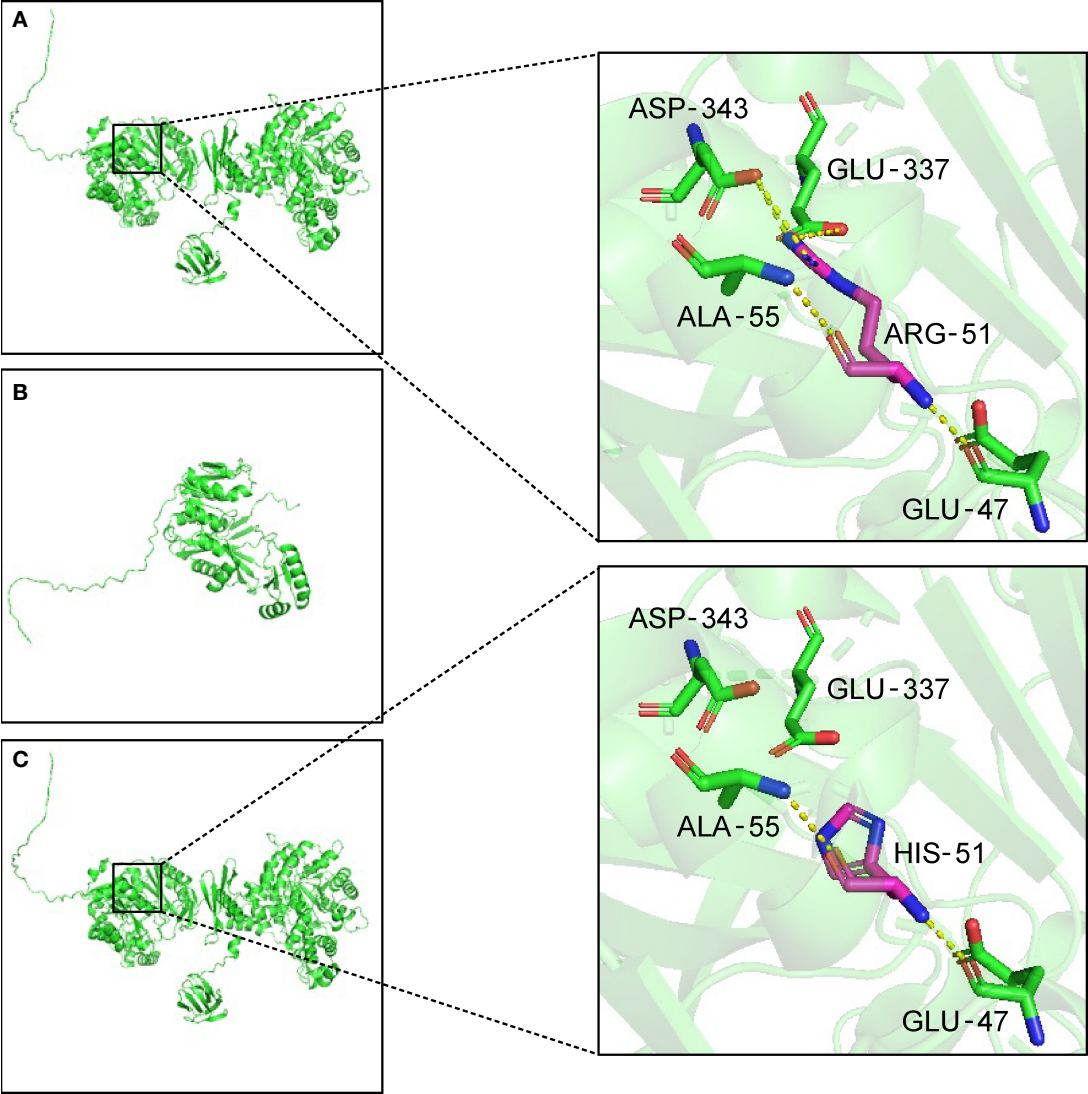
Three-dimensional structure of the PC protein. **(A)** In wild-type protein, ARG-51 forms one hydrogen bond each with GLU-47 and ALA-55, and two hydrogen bonds each with GLU-337 and ASP-343. **(B)** Maternal mutant proteins of the variant c.1154_1155del. The frameshift mutation results in the premature termination of protein translation and the alteration of three-dimensional structure. **(C)** Paternal mutant protein of the variant c.152G>A. After the mutation, the hydrogen bonds are broken between HIS-51 and GLU-337 and ASP-343. The yellow dotted lines indicate hydrogen bonds. ALA, Alanine. ARG, arginine. ASP, aspartic acid. GLU, glutamic acid. HIS, histidine.

Trio-WES analysis detected a compound heterozygous mutation in the PC gene of the proband. c.1154_1155del (p.Arg385GlnfsTer10), Exon11/23, was identified in the patient’s asymptomatic mother and c.152G>A (p.Arg51His), Exon5/23, was identified in the patient’s asymptomatic father. The maternal mutation was a frame-shift mutation and this variation has not been recorded in the reference population Genome Aggregation Database (gnomAD) for minimum allele frequency. The variation was reported as Likely Pathogenic in the Clinvar database (https://www.ncbi.nlm.nih.gov/clinvar/variation/1184451). Analysis showed that the heterozygous mutation was considered a pathogenic variation according to American College of Medical Genetics and Genomics (ACMG) guidelines (PVS1+PM2_Supporting+PP4). The paternal mutation is a missense mutation and the variation had a minimum allele frequency of 0 in the gnomAD. Multiple predicted results (SIFT, http://sift.jcvi.org: D. Polyphen2_HDIV and HVAR, http://genetics.bwh.harvard.edu/pph2/index.shtml: D. REVEL, https://sites.google.com/site/revelgenomics/: 0.928) suggested that the mutation had a high probability of harmful effects on protein structure or function. The mutation was not reported in the Clinvar database and was considered a Likely Pathogenic mutation according to ACMG guidelines (PM2_Supporting+PM3+PP3_Moderate+PP4). The SWISS-MODEL (https://www.swissmodel.expasy.org/) and PyMOL software (https://pymol.org/2/) were used to predict and compare the spatial structure of the wild-type protein and mutant protein. Compared to the tertiary structure of wild-type proteins (**Figure 1A**), the c.1154_1155del mutation caused the 385th arginine to become glutamine and the 10th downstream amino acid to become termination codon, which resulted in structural change and loss of function of the protein (**Figure 1B**). The c.152G>A mutation led to the 51st arginine to be replaced by histidine, which failed to form hydrogen bonds with the 343rd aspartic acid and the 337th glutamic acid. The breakdown of four hydrogen bonds may result in the instability of the protein’s local structure and thus affect the its function (**Figure 1C**). Furthermore, the preimplantation genetic testing for monogenic disorders (PGT-M) can prevent the couple from having a second child with the mutant PC gene, so further verification of mutation site information in pedigree and single nucleotide polymorphism (SNP) linkage analysis were performed as displayed in **Supplementary Figures 1**, **2**.

At 12 weeks of gestation, the thickness of fetal nuchal translucency was 0.7mm measured by fetal ultrasound. At 22w5d’ gestation, ultrasound images displayed that there were small cysts in the anterior horns of the lateral ventricle, and the posterior horns of the lateral ventricle were enlarged. Moreover, increased biparietal diameter and head circumference were detected on fetal ultrasonography during the whole pregnancy. According to the National Institute of Child Health and Human Development (NICHD) Fetal Growth Curve (Asian) (12), biparietal diameter and head circumference at different gestational weeks were generally above the 90th or even 95th percentile (**Supplementary Table 1**).

**Figure 2:**
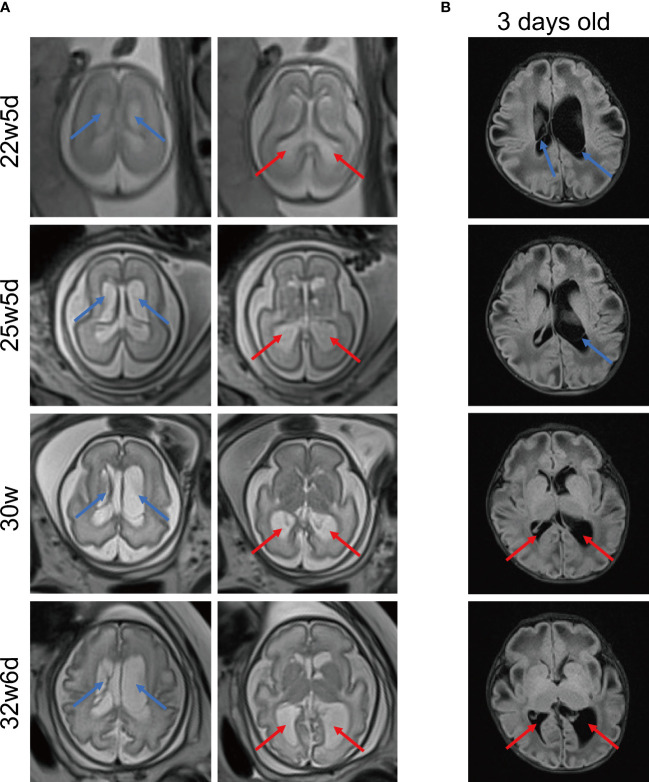
Representative MRI images of brain. **(A)** MRI images of fetal brain at different gestational ages. MRI T2 HASTE sequence, transverse view. **(B)** Brain MRI images of the 3-day-old newborn. MRI T2 FLAIR sequence, transverse view. The blue arrows indicate multiple subependymal cysts and the red arrows indicate enlargement of posterior horns of lateral ventricles. MRI, magnetic resonance imaging. HASTE, half-fourier acquisition single-shot turbo spin echo. FLAIR: fluid-attenuated inversion recovery.

Due to the aberrant brain structure indicated by ultrasound in the pregnant mother at 22w5d’ gestation, a fetal brain MRI was performed. MRI images (**Figure 2A**) displayed that long T2 signals were seen near the anterior horns and bodies of bilateral lateral ventricles, which may indicate subependymal cysts. In addition, bilateral posterior horns of lateral ventricles were widened. Subependymal cysts and lateral ventricular widening persisted throughout the pregnancy. As the gestational age increased, these brain abnormalities were more pronounced, especially on the left side. The neurosurgeon evaluated that the fetal brain development was normal and there was no hydrocephalus. As shown by **Figure 2B**, multiple and large subependymal cysts were still visible after this patient was born, the largest of which exceeded 4cm in long diameter. Meanwhile, the posterior horn of the left lateral ventricle was severely widened to more than 2 cm.

When ultrasound and MRI revealed brain structure abnormalities, amniocentesis was performed immediately according to the doctor’s recommendation. The results showed that the fetal chromosome karyotype was 46, XX. Copy number variation analysis revealed a 0.64MB microdeletion on chromosome 10 (chr10:g.47080000_47720000del), rated as Likely Benign. Unfortunately, further WES was not performed.

## 3 Discussion

This patient experienced a 10-fold increase in lactic acid levels within hours of birth, accompanied by metabolic acidosis, hyperammonemia, hypercitrullinemia, progressively elevated bilirubin, and elevated urinary organic acids. Eventually, she survived for only 26 days. Based on clinical features and genetic analysis, she was diagnosed with type B PCD (13, 14). Recently, a PCD type B newborn with similar manifestations died on the 22nd day after birth (9). Knowledge of PCD is inadequate in China. So far, this patient is the fourth case of PCD and the first case of type B reported in the Chinese population. The three previous cases were all confirmed as PCD type A and two of them died at about 1 and 3 years of age, respectively (2–4). Consistent with previous reports, a fatal outcome in the neonatal period was observed in patients with PCD type B, while most patients with type A could survive to infancy or early childhood (5, 9).

PC gene dysfunction is passed on to offspring in an autosomal recessive manner (1). By genetic analysis, a compound heterozygous variant was identified in the PC gene. The paternal mutation is a novel and pathogenic variant that hasn’t been reported in the previous literature. Therefore, this report enriches the pathogenicity variation and phenotype spectrum of the PC gene. In addition to genetic diagnostic tools, direct determination of PC activities in the chorionic villi or cultured amniotic fluid cells in the presence of family history can help prenatally diagnose PCD (15, 16). However, when a non-consanguineous couple conceives their first child, PCD is not easily and accurately identified in the prenatal stage. PC is not only a key enzyme for gluconeogenesis, but also plays an anaplerotic role in the brain, such as neurotransmitter synthesis, which supports the essential role of PC in brain development (7). Hence, the fetal unusual neuroradiological presentations indicated by ultrasound and MRI were retrospected to provide clues for prenatal diagnosis of PCD.

Neuroimaging frequently disclosed aberrant periventricular white matter signals, widened lateral ventricles, and large and multiple cysts in PCD patients, which was associated with energy deprivation induced by PC deficiency (5, 7). Clinically, the neurological disorders of PCD vary depending on the type and severity of the disease. PCD type A and type B usually present with developmental delay, hypotonia, seizures, and abnormal movements such as rigidity and hypokinesia (5, 7). Mild neurological impairment in PCD type C simply manifests as a developmental delay with relatively long survival (17). But type C also rarely caused acute flaccid paralysis (11). Evidence suggests that the brain lesions in patients with PCD started prenatal (18, 19). Brun first documented periventricular leukomalacia by ultrasonography at 29.4 weeks of gestation (20). MRI images obtained from PCD patients at 10 days of age showed abnormal white matter signal, immature gyral pattern, large periventricular cysts, and dilatation of the occipital and temporal horns, before any metabolic decompensation. However, we observed these aberrant brain structures much earlier, at 22 weeks of gestation. Furthermore, larger head circumference and biparietal diameter measured by ultrasound may be another prominent feature of fetal PCD. Twins with PCD had increased head circumference in the fetal stage and macrocephaly at birth (20). In this case’s fetal ultrasound examination, her head circumference and biparietal diameter were consistently at the high percentile level. Infrequently, brain damage caused by hyperammonemia cannot be ignored, especially in type B PCD. The level of ammonia in a Turkish PCD patient unexpectedly elevated to 860μM, resulting in hyperammonemic encephalopathy (21).

The manifestations of fetal brain injury in this case need to be distinguished from Vein of Galen Malformations (VGAM), which may be characterized by intracranial cystic mass and hydrocephalus. However, VGAM is usually accompanied by congestive heart failure, hence fetal cardiomegaly, fetal edema, and blood flow signals in the intracranial cyst can be detected by ultrasound (22).

There is currently no specific treatment for PCD patients. Hydration therapy and correction of metabolic acidosis are critical during acute decompensation. Patients can receive supplementation with co-factors and end-products involved in the metabolism of pyruvate, such as thiamine, biotin, citrate, aspartic acid, and lipoic acid (5, 9). Neurological treatment includes the use of antiepileptic drugs (5). As for neurosurgery, if infants had communicating and non-communicating hydrocephalus or ventricle enlargement with neurodevelopmental abnormalities, ventriculoperitoneal shunt could be performed and then careful postoperative fluid management should be paid attention to (23, 24).

In conclusion, we present a newborn girl who presented with tachypnea and restlessness with refractory lactic acidosis. She was diagnosed with PCD type B by biochemical tests and genetic sequencing. We report the earliest onset of prenatal brain lesions in the second trimester. If the fetuses manifest brain abnormalities such as subependymal cysts, aberrant white matter signals, and lateral ventricle widening during pregnancy, it is necessary to perform gene sequencing using amniotic fluid for genetic metabolic diseases as soon as possible, which may be beneficial for early diagnosis of the disease.

## Data availability statement

The datasets presented in this study can be found in online repositories. The names of the repository/repositories and accession number(s) can be found below: https://www.ncbi.nlm.nih.gov/bioproject/PRJNA979804.

## Ethics statement

The studies involving human participants were reviewed and approved by Ethics committee of Zhongnan Hospital of Wuhan University. Written informed consent to participate in this study was provided by the participants’ legal guardian/next of kin. Written informed consent was obtained from the individual(s), and minor(s)’ legal guardian/next of kin, for the publication of any potentially identifiable images or data included in this article.

## Author contributions

MX collected the data and wrote the manuscript.
