# Supplementary material for: Case Report: Prenatal neurological injury in a neonate with pyruvate carboxylase deficiency type B
Source: Front Endocrinol (Lausanne). 2023 Jul 7;14:1199590. doi: 10.3389/fendo.2023.1199590 (PMC10360024; doi:10.3389/fendo.2023.1199590)
Supplement: Supplementary file 1 [file DataSheet_1.docx]

Supplementary Material

Case report: Prenatal neurological injury in a neonate with pyruvate carboxylase deficiency type B

Mei Xue*

*** Correspondence:** Mei Xue: xuem1993@163.com

# Supplementary Materials and methods

**Whole exome sequencing**

The genomic DNA was extracted from the patient's and her parents' peripheral blood using QIAamp Blood Genomic DNA Extraction Kit according to the manufacturer's protocol. The concentration and purity of DNA were detected by a NanoDrop 2000. DNA was sheared into proper pieces (150-200 bp) by a Covaris M220 focused-ultrasonicator and fragments of DNA were amplified after terminal repair and added to the reaction. The exome sequencing kit (xGen® Exome Research Panel; Integrated DNA Technologies, Inc.) was used to capture the amplified library. PE150 (Paired-end 150 bp) sequencing was performed on an Illumina Novaseq 6,000.

# Supplementary Figures

## Supplementary Figures 1
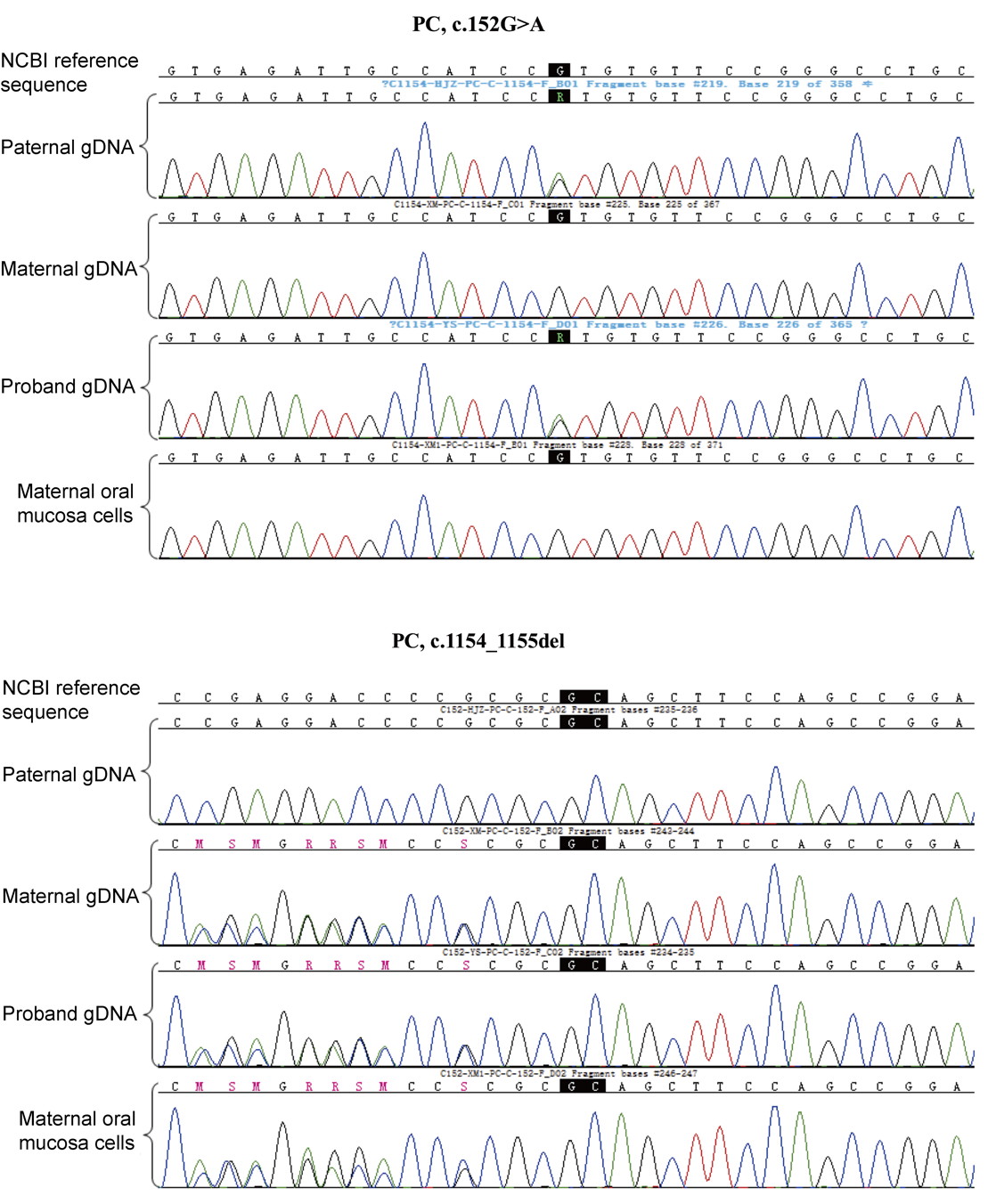


**Supplementary Figure 1.** Verification of mutation site information in pedigree. The sequencing map converts the reverse sequencing result into the standard sequence.

## Supplementary Figures 2


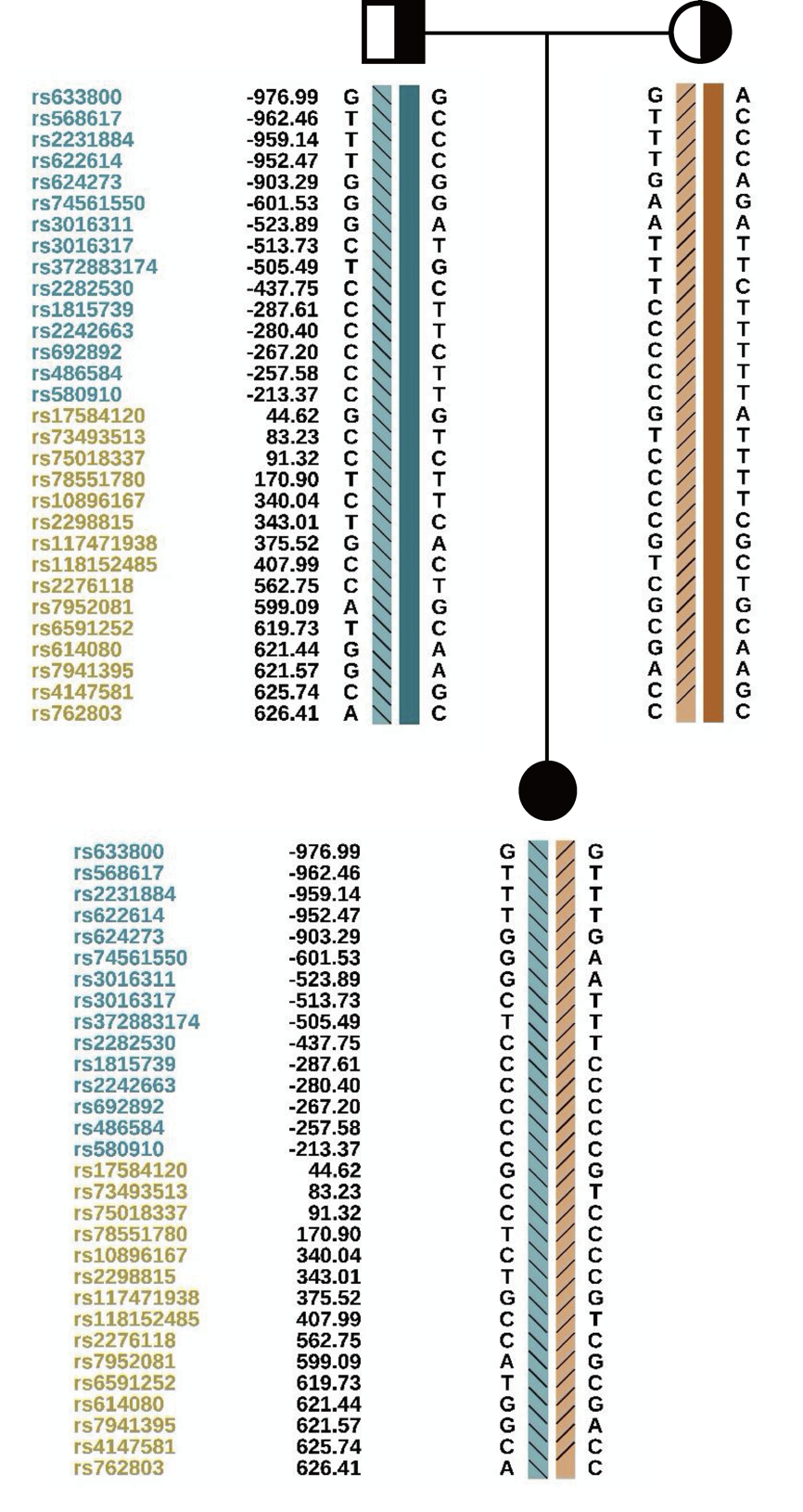


**Supplementary Figure 2.** Family genetic pedigree and SNP linkage analysis. The proband’ parents were asymptomatic carriers of the mutation. SNP, single nucleotide polymorphism.

# Supplementary Tables

## 3.1 Supplementary Table 1

Supplementary Table 1. Fetal biparietal diameter and head circumference measured by ultrasound.

| Gestational weeks | Biparietal diameter (cm)  (Percentile) | Head circumference (cm)  (Percentile) |
| --- | --- | --- |
| 22w5d | 5.97 (94.3%) | 21.07 (83.9%) |
| 25w5d | 7.11 (98.2%) | 24.78 (90.9%) |
| 32w | 8.99 (99%) | 31.62 (98.3%) |
| 34w1d | 9.4 (98.4%) | 32.77 (95.3%) |
| 36w5d | 9.67 (95.6%) | 34.13 (93.5%) |
| 38w5d | 10.16 (98.7%) | 34.49 (87.5%) |

The percentile was calculated according to National Institute of Child Health and Human Development (NICHD) Fetal Growth Curve (Asian).
